# Burosumab for X-linked Hypophosphataemia and Tumour-induced Osteomalacia in Adults

**DOI:** 10.17925/EE.2026.22.1.6

**Published:** 2026-05-26

**Authors:** Simon Black, Richard Keen, Judith Bubbear

**Affiliations:** Metabolic Bone Disease Centre, Royal National Orthopaedic Hospital, Royal National Orthopaedic Hospital, Brockley Hill, Stanmore, Middlesex, UK

**Keywords:** Burosumab, fibroblast growth factor (FGF)-23, hypophosphataemia, osteomalacia, rickets, tumour-induced osteomalacia, X-linked hypophosphataemia

## Abstract

X-linked hypophosphataemia and tumour-induced osteomalacia are rare, distinct metabolic conditions that can affect both children and adults, and result in elevated fibroblast growth factor-23 levels and subsequent low phosphate levels. This leads to severe musculoskeletal symptoms and complications, and these conditions carry significant disease burden and high levels of morbidity. In this article, we examine the conditions and analyse the use of burosumab, a recently developed monoclonal antibody treatment, in the care of adults with these conditions.

## Pathophysiology of X-linked hypophosphataemia

X-linked hypophosphataemia (XLH) is a progressive skeletal disorder, originally defined by Albright in 1937 as hypophosphataemic vitamin D rickets.^1^ XLH is the commonest form of inherited rickets/osteomalacia and is an X-linked dominant condition.^2^ The most common mutation is a loss-of-function mutation in PHEX (phosphate-regulating endopeptidase homologue X-linked), a phosphate-regulating gene with homologies to endopeptidases on the X-chromosome. PHEX is expressed in osteocytes and odontoblasts and regulates fibroblast growth factor-23 (FGF-23) gene expression and production. Elevated FGF-23 levels and excess activity occur due to the PHEX mutation.^3^

FGF-23 causes down-regulation of surface expression of sodium-phosphate co-transporters NPT2a and NPT2c, leading to renal phosphate wasting. The PHEX enzyme ordinarily degrades and removes osteopontin, an inhibitor of bone mineralization, and, therefore, PHEX deficiency also causes accumulation of osteopontin. Hypophosphataemia particularly affects the growth plate chondrocytes and osteoid surfaces, resulting in impaired hypertrophic chondrocyte apoptosis and reduced mineral apposition rates.^4^

## Epidemiology and presentation

Prevalence of XLH is thought to be 1.7 per 100,000 children to 4.8 per 100,000 persons (children and adults).^5^ Symptoms usually develop in childhood, together with delay in walking, lower limb deformities and slow skeletal growth. Dental abnormalities can also occur. In severe childhood cases, craniosynostosis, frontal bossing, Chiari malformations and hearing loss can also occur. There is a predilection for the condition to affect the long bones of the lower limbs, and short stature can ensue, which can be exacerbated during puberty.

In adulthood, XLH can present with fractures, pseudofractures and early-onset osteoarthritis. Bony or joint pain is a universal symptom across all ages; the vast majority of patients will have established osteomalacia. Fatigue and joint stiffness are also common. Enthesitis, ligament calcification and significant dental disease can also occur. A summary of the signs and symptoms in XLH can be seen in *Table 1*. XLH leads to significant reductions in mobility and, hence, has a profound impact on daily functioning and quality of life, with subsequent impact on employment, mental health and general physical wellbeing. Indeed, burdens of XLH persist throughout the patient’s lifespan and encompass physical, social and emotional aspects; these are often compounded by lack of access to specialist care and treatment.^6^

Hypophosphataemia on serum bloods is the hallmark biochemical abnormality, together with phosphaturia and a low or low-normal 1,25-hydroxycolecalciferol (1,25[OH]_2_) level. Corrected calcium and parathyroid hormone levels are usually normal. Renal phosphate wasting can be assessed through measurement of tubular maximal reabsorption of phosphate, adjusted for the glomerular filtration rate (TmP/GFR), or the percentage tubular reabsorption of phosphate.

**Table 1:** Main signs and symptoms of X-linked hypophosphataemia

Sign	Symptom
Muscle deficits	Poor muscle development
	Poor muscle function
	Poor muscle quality
	Muscle weakness
	Muscle fatigue
Joint deficits	Early-onset osteoarthritis
	Enthesopathy
	Spinal stenosis
Bone deficits	Short stature
	Poor bone quality
	Osteomalacia
	Fractures/pseudofractures
	Osteophyte formation
Other deficits	Dental defects
	Hearing loss

## Conventional therapy for X-linked hypophosphataemia

Conventional treatment of XLH involves oral phosphate replacement supplementation, often in large doses of 750–1,600 mg daily.^7^ High doses of active vitamin D supplements would also be given, often calcitriol or alfacalcidol, at doses of 0.50–0.75 and 0.75–1.5 μg, respectively.

Oral phosphate supplements can commonly lead to gastrointestinal side effects.^8^ In addition, oral phosphate and active vitamin D can promote hyperparathyroidism and cause nephrocalcinosis. Regular biochemical monitoring and interval imaging are required when patients are on conventional therapy, as outlined in the recent guidelines by Haffner et al.^6^ With conventional therapy involving oral phosphate and vitamin D supplementation, response to therapy is assessed by significant improvement in musculoskeletal pain and stiffness, improvement in pseudofractures or other radiological rickets lesions and normalization of total or bone-specific alkaline phosphatase.^9^ Serum phosphate levels remaining low despite oral supplementation are not a target for adjusting therapy. With burosumab therapy, significant improvement in renal phosphate wasting, serum phosphate levels and musculoskeletal pain is assessed at 6 months, in addition to the same factors as with conventional treatment being assessed at the 12-month point.

## Burosumab for X-linked hypophosphataemia

Burosumab is a fully humanized immunoglobulin-G1 (IgG1) monoclonal antibody that binds to FGF-2 3 and inhibits its activity.^10^ It is licensed for the treatment of XLH at a dose of 0.8–1.2 mg/kg every 2 weeks in children and 1 mg/kg subcutaneous injections every 4 weeks in adults. Dosing can be titrated according to serum phosphate levels, with an aim to maintain them within the low-to-mid normal range. Side effects are uncommon, although injection-site reactions and restless legs have been reported in approximately 17% of patients.^11^

In children with XLH, four studies since 2018 have shown burosumab to lead to sustained improvement in phosphate levels and metabolism, together with decreased pain scores over a period of up to 160 weeks. Radiographic improvement up to 40 weeks has also been demonstrated in established rickets.^12^

Use of burosumab in adults was first evaluated in a double-blind, randomized, placebo-controlled study of 38 patients.^13^ In this study, increasing doses of burosumab were given with no other supplement-based treatment. Primary endpoints of TmP/GFR, serum phosphate and serum 1,25(OH)_2_-vitamin D levels were used, all of which showed improvement. Nausea (24%) and headache (18%) were the most commonly reported adverse effects, but no significant safety issues were identified. *Table 2* contains a summary of adult clinical trials involving burosumab for the treatment of X-linked hypophosphataemia.^11,13–18^

The first large, international, multi-centre, phase III trial of burosumab was published by Insogna et al. in 2018.^14^ In this double-blind, randomized controlled study, 134 adults (aged 18–65 years) were identified across the USA, France, UK, South Korea, Ireland, Italy and Japan; all of whom were confirmed to have the PHEX mutation or had met criteria including serum phosphate below 0.81 mmol/L and TmP/GFR below 2.5 mg/dL. Burosumab was administered subcutaneously every 4 weeks over a 24-week period. Increased renal phosphate reabsorption and normalized serum phosphate levels were achieved throughout the dosing intervals, as well as increased serum 1,25(OH)_2_-vitamin D levels. In this study, more than half of the patients were found to have active fractures or pseudofractures on radiological skeletal survey at baseline due to biochemical osteomalacia, despite previous treatments with oral supplementation. Use of burosumab during the trial period was found to have resulted in full healing of nearly half of those fractures identified, highlighting the positive benefits in resolving clinical signs of osteomalacia as well as biochemical abnormalities. An interesting observation in this study was that the mean body mass index of the patients was over 30 kg/m², highlighting the effect of XLH on physical activity, mobility, general disease burden and subsequent morbidity. The trial also assessed stiffness as a primary endpoint using the Western Ontario and McMaster Universities Osteoarthritis Index (WOMAC) score. Whilst acknowledging that there are multi-factorial reasons contributing to stiffness as a symptom, burosumab demonstrated statistically significant reductions in stiffness, postulated as being due to improvements in muscle functioning from normalization of phosphate. Whilst pain and physical function improved, these were not statistically significant results. Burosumab also demonstrated a satisfactory safety profile.

Whilst the Insogna trial assessed results over the first 24 weeks of treatment with burosumab, Portale et al. subsequently documented findings from the subsequent week 24–48 open-label period of the same patient cohort.^14,15^ During this period, all patients received burosumab, including those previously given placebo treatment. Serum phosphate levels remained normal in nearly 84% of those who had received burosumab throughout the trial and became normal in 89% of those initially in the placebo arm. The proportion of fully healed fractures and pseudofractures progressively increased between weeks 24 and 48. Stiffness and functional exercise capacity, as measured by the 6 min total distance walk test, were found to have improved at statistically significant levels. This study, therefore, highlighted the on-going and progressive clinical benefits of burosumab treatment over longer periods.

A smaller study of 11 patients was also completed by Insogna et al. in 2019, analysing improvements in osteomalacia specifically in patients who were previously completely untreated in the 2-year period prior to enrolment in the trial.^19^ This looked at histomorphometric measurements, predominantly of osteoid volume/bone volume, via transiliac bone biopsies at baseline and at 48 weeks, in addition to serum phosphate levels and fracture healing. Once again, subcutaneous doses of 1 mg/kg burosumab were administered every 4 weeks.

**Table 2:** Summary of adult clinical trials involving burosumab for X-linked hypophosphataemia and tumour-induced osteomalacia^11,13–18^

Study	Study design	Population	Burosumab dose/administration	Outcomes
Carpenter et al.^13^	Phase I double-blind, placebo-controlled, randomized	Adults with XLH (n = 38)	0.003–003–0.3 mg/kg IV or 0.1–1 mg/kg SC single dose (versus placebo)	Increased TmP/GFR, serum Pi and serum 1,25(OH)_2_D
Imel et al.^16^	Phase I/II open-label, dose escalation	28 adults with XLH; 22 in 12 month extension study	0.05, 0.1, 0.3, 0.6 mg/kg SC escalating every 4 weeks	Increased TmP/GFR, serum Pi and 1,25(OH)_2_D
Carpenter et al.^17^	Phase II open-label (single-arm, dose-finding)	16 adults with TIO	0.3–3–2.0 mg/kg SC every 4 weeks	Increased TmP/GFR, serum Pi and 1,25(OH)_2_-vitamin D; one serious adverse event
Insogna et al.^14^	Phase III double-blind, placebo-controlled, randomized	134 adults with XLH, confirmed PHEX mutation	1 mg/kg SC every 4 weeks (versus placebo)	Improved WOMAC stiffness subscale, but not some other measures; acceptable safety profile
Portale et al.^15^	Phase III extension – open-label period	Same cohort as Insogna et al.^11^	1 mg/kg SC every 4 weeks	Enabled maintenance of normal serum Pi; increase in healed fractures; improved physical outcome measures
Insogna et al.^19^	Phase III double-blind, placebo-controlled, randomized	11 patients with XLH with paired biopsies	1 mg/kg SC every 4 weeks (versus placebo)	Improved osteomalacia as measured by bone histomorphometry
Cheong et al.^18^	Phase I open-label, dose escalation	15 patients with XLH	0.3 versus 0.6 versus 1.0 mg/kg SC single dose	Increased TmP/GFR, serum Pi and 1,25(OH)_2_D; no serious adverse events

*IV = intravenous; PHEX = phosphate-regulating endopeptidase homologue X-linked; Pi = inorganic phosphate; SC = subcutaneous; TIO = tumour-induced osteomalacia; TmP/GFR = tubular maximal reabsorption of phosphate, adjusted for the glomerular filtration rate; WOMAC = Western Ontario and McMaster Universities Osteoarthritis Index; XLH = X-linked hypophosphataemia.*

All histomorphometric measurements (osteoid volume/bone volume, osteoid thickness, osteoid surface/bone surface and mineralization lag time) improved significantly over 48 weeks, together with increases in levels of bone formation and resorption markers. All of these four key histomorphometric measurements were elevated at baseline in all of the patients, emphasizing the presence of profound underlying osteomalacia in untreated XLH patients. Additionally, healing rates of fractures and pseudofractures were similar to those seen in previous studies with burosumab treatment.^16^

Conventional supplementation regimens for treatment of XLH have consistently demonstrated hypercalciuria, tertiary hyperparathyroidism and nephrocalcinosis as adverse outcomes.^20^ Trials of burosumab therapy have, conversely, consistently shown no changes in parathyroid hormone levels, calcium concentrations nor nephrocalcinosis scores, or evidence of this on ultrasound.^9,14^ Nevertheless, renal tract ultrasound is still recommended for monitoring in current clinical use.^9^

Treatment with burosumab in adult patients with XLH has now become further established in the UK, with the recently updated National Institute for Health and Care Excellence (NICE) guidance in August 2024 recommending its use. NICE guidance on the use of burosumab in children was published in 2018 and recently revised in April 2025.^21^ The European Commission approved its use in 2020.^22^ Nevertheless, there remains scope for further evaluation of its impact. Notably, there has not been any head-to-head data directly comparing the use of burosumab with, or in addition to, conventional supplementation treatments. There is also potential to assess the benefits of burosumab on other clinical manifestations of XLH, such as dental symptoms and loss, craniosynostosis, need for orthopaedic surgeries and arthropathies. In other countries, access may be limited to adults who have not responded to conventional therapies.

## Pathophysiology or tumour-induced osteomalacia

Tumour-induced osteomalacia (TIO) is a paraneoplastic phenomenon caused by phosphaturic mesenchymal tumours that secrete FGF-23.^17^ This causes decreased renal proximal tubular reabsorption of phosphate, resulting in a chronic hypophosphataemic state. Low phosphate, combined with clinical features of widespread bone pain, fragility fractures and muscle weakness, leads to the diagnosis; however, this is frequently delayed due to the rarity of the condition and the often non-specific presentation. The tumours are usually very small, leading to difficulty in localizing them, and surgical excision remains the definitive treatment.^23^

FGF-23 levels are usually elevated, but can remain inappropriately normal, and circulating levels of 1,25-dihydroxyvitamin D3 are either low or low-normal.^18^ Two fusion genes coding for fibronectin, FN1-FGFR1 and FN1-FGF1, are implicated in the development of the condition and are thought to be present in approximately half of the mesenchymal tumours, which tend to grow slowly and can present anywhere in the body. The condition is rare in those under the age of 18.

Renal phosphate wasting due to excess FGF-23 is the pathophysiological mechanism in TIO. This leads to inefficient bone mineralization and osteomalacia, causing reduced bone mineral density, disrupted bone microarchitecture and can lead to insufficiency fractures. TIO therefore has a high morbidity and disease burden, with chronic pain, weakness, fatigue, reduced mobility and reduced quality of life scores.^24^

Parathyroid hormone (PTH) and FGF-23 are thought to suppress the expression of type 2a and 2c sodium-phosphate co-transporters, and this leads to inhibition of proximal tubular phosphate reabsorption.^25^

Low vitamin D and high-phosphate diets are also thought to increase production and levels of circulating FGF-23.^24^ Many other factors, including PTH, cytokines, erythropoietin, iron deficiency, sclerostin, calciprotein particle, lipocalin-2, aldosterone and myostatin, can be implicated in increasing FGF-23.^26–28^

Phosphaturic mesenchymal tumours were only established as a defined histological entity in 2004 by Folpe et al., who described tumours composed of round-oval spindle cells together with florid vascularization and excess extracellular matrix that calcifies.^29^ The tumours can easily infiltrate into surrounding tissues, thus making resection difficult. In bone, the tumours can infiltrate intratrabecular spaces and produce an abundant osteoid-like matrix, resembling an osteosarcoma.^29^ Whilst the tumours are usually solitary and benign, they can be multiple and metastasize, where they have been found to have high cellularity, necrosis, high mitotic activity, high Ki67 indices (a nuclear protein in dividing cells used as a marker for cell proliferation) and high levels of p53 expression (a tumour suppressor gene that stops tumour formation).

## Epidemiology and presentation

There have been fewer than 1,000 reported cases of TIO in the literature, but incidence has been reported as 0.70 per 100,000 persons (0.43 per 100,000 adults).^30^ Bone pain is a common, albeit non-specific, finding in patients with TIO, and symptoms are often unrelated to the site and size of the tumour.^28^ Delays in diagnosis are common and can lead to presentation after many years, with severe generalized weakness and well-established bony damage and severe spinal deformity.^31^ Presentations in childhood cases can have associated rickets. Rarely, subcutaneous corresponding lumps can be palpable if the tumour resides in particular muscular regions. Plain X-ray imaging will show evidence of osteopaenia with coarse trabecular bone patterns, Looser’s zones and bowing of the long bones. Bone mineral density is commonly reduced, and there may be evidence of insufficiency fractures.^32^

Persistently low phosphate levels, together with low or inappropriately normal 1,25-dihydroxyvitamin D levels, are hallmarks of the condition.^28^ Calculation of the TmP/GFR is required to ascertain the extent of renal tubular phosphate wasting. This will be reduced in TIO (normal range is approximately 2.5–4.5 mg/dL).^33^ FGF-23 levels will be elevated, although there are various assays available to measure this, with no clear evidence regarding the most accurate.

The lower extremities are the most common site for mesenchymal tumours associated with TIO, followed by the head and neck, torso and then the upper extremities.^34^ Various imaging modalities have been trialled in the investigation of TIO.^35^ Initially, this involved the use of octreotide combined with single-photon emission computerised tomography (SPECT) of the whole body to identify lesions. Use of a technetium-99m-labelled somatostatin analogue has been found to have excellent specificity of >99%; however, the gold-standard investigation is felt to be with DOTA-TATE scanning, whereby a Gallium-68 positron-emitting radionuclide linked to a chelator (DOTA or tetraxetan) is joined to tyrosine-3-octreotate.^36^ These scans have the greatest sensitivity, with a high affinity for SST2 (somatostatin-2) substrates that are expressed on the cell surface of offending tumours.

## Treatment of tumour-induced osteomalacia

Complete surgical resection of causative tumours is the curative treatment for TIO; however, identification and localization of tumours is often difficult. Incomplete resection will lead to recurrence. It is thought that 35–40% of tumours cannot be localized.^37^

When surgery is not a treatment option, multiple doses of oral phosphate and active vitamin D analogue supplementation were required.^18^ These can commonly cause gastrointestinal upset and symptoms, together with calcium and parathyroid hormone imbalances.

## Evidence of burosumab in tumour-induced osteomalacia

Use of burosumab for the treatment of TIO has recently been described and a summary of the clinical trials are included in Tablet 2: Imanishi et al. first described a study of 13 patients in Japan and South Korea with TIO in 2021, whereby burosumab was initiated at a dose of 1 mg/kg and subsequently up-titrated depending on the individual’s phosphate levels.^38^ The optimal dose of burosumab in this study could not be defined due to wide variation between patients. The study confirmed the ability of burosumab to increase the key endpoints of serum phosphate levels, serum 1,25(OH)_2_-vitamin D levels and TmP/GFR between weeks 14 and 112. Treatment with burosumab was also evaluated by clinical improvement endpoints of greater ability to walk further, as defined by 6 min walk testing, decreased patient-reported pain levels and evidence of healing of fractures and pseudofractures.

Jan De Beur et al. describe a similarly sized concurrent trial in the USA assessing endpoints of serum phosphate and osteomalacia, as assessed by transiliac bone biopsies performed at week 48.^39^ As well as normalizing phosphate levels, burosumab was found to improve osteoid volume and thickness and reduce mineralization lag time. Widespread reduction in patient-reported levels of both pain and fatigue was found once established on burosumab treatment, and an acceptable safety profile was described, with mild-to-moderate adverse effects reported.

In addition to XLH, burosumab therefore represents a potentially valuable and effective treatment in TIO where resection or localization of tumours is not possible or successful. Further studies are required to assess long-term use in controlling symptoms; however, there is evidence to currently support the use of burosumab in improving patient quality of life scores, reducing longer term disease sequelae and minimizing concerns surrounding safety.

## Conclusion

Burosumab, a novel monoclonal antibody, represents a significant advancement in the treatment of both XLH and TIO, conditions that have previously carried significant levels of disease burden and morbidity. Previous treatments have relied upon cumbersome supplementation regimens, with only partial benefits in terms of symptom relief, disease progression and quality of life. Treatment with burosumab is now established in both children and adults with these conditions, with proven benefits as outlined in this article. Prompt diagnosis of the conditions, with access to specialist care, and prompt commencement of the drug, are now key to further successful treatment.

## References

[1] Albright F, Butler AM, Bloomberg E (1937). RICKETS resistant to vitamin d therapy.. Arch Pediatr Adolesc Med..

[2] Baroncelli GI, Mora S (2021). X-linked hypophosphatemic rickets: Multisystemic disorder in children requiring multidisciplinary management.. Front Endocrinol (Lausanne)..

[3] Watts C, Hall M, Watts R (2009). Oxford Desk Reference: Rheumatology..

[4] Imel EA (2021). Burosumab for pediatric x-linked hypophosphatemia.. Curr Osteoporos Rep..

[5] Giannini S, Bianchi ML, Rendina D (2021). Burden of disease and clinical targets in adult patients with x-linked hypophosphatemia. A comprehensive review.. Osteoporos Int..

[6] Cheung M, Rylands AJ, Williams A (2021). Patient-reported complications, symptoms, and experiences of living with x-linked hypophosphatemia across the life-course.. J Endocr Soc..

[7] Haffner D, Emma F, Eastwood DM (2019). Clinical practice recommendations for the diagnosis and management of x-linked hypophosphataemia.. Nat Rev Nephrol..

[8] BNF. Side Effects. On. http://NICE.org.uk.

[9] Haffner D, Emma F, Seefried L (2025). Clinical practice recommendations for the diagnosis and management of x-linked hypophosphataemia.. Nat Rev Nephrol..

[10] NICE. (2024.). Burosumab for treating X-linked hypophosphataemia in adults: Technology appraisal guidance.. http://www.nice.org.uk/guidance/ta993.

[11] Schindeler A, Biggin A, Munns CF (2020). Clinical evidence for the benefits of burosumab therapy for x-linked hypophosphatemia (xlh) and other conditions in adults and children.. Front Endocrinol..

[12] Linglart A, Imel EA, Whyte MP (2022). Sustained efficacy and safety of burosumab, a monoclonal antibody to FGF23, in children with x-linked hypophosphatemia.. J Clin Endocrinol Metab..

[13] Carpenter TO, Imel EA, Ruppe MD (2014). Randomized trial of the anti-FGF23 antibody KRN23 in x-linked hypophosphatemia.. J Clin Invest..

[14] Insogna KL, Briot K, Imel EA (2018). A randomized, double-blind, placebo-controlled, phase 3 trial evaluating the efficacy of burosumab, an anti-FGF23 antibody, in adults with x-linked hypophosphatemia: Week 24 primary analysis.. J Bone Miner Res..

[15] Portale AA, Carpenter TO, Brandi ML (2019). Continued beneficial effects of burosumab in adults with x-linked hypophosphatemia: Results from a 24-week treatment continuation period after a 24-week double-blind placebo-controlled period.. Calcif Tissue Int..

[16] Imel EA, Zhang X, Ruppe MD (2015). Prolonged correction of serum phosphorus in adults with x-linked hypophosphatemia using monthly doses of KRN23.. J Clin Endocrinol Metab..

[17] Carpenter TO, Weber MP, Peacock T (2016). Effects of KRN23 (anti-FGF23 antibody) in patients with tumour-induced osteomalacia and epidermal naevus syndrome: Results from an ongoing phase 2 study.. J Bone Miner Res..

[18] Cheong HI, Yoo H, Adachi M (2019). First-in-Asian phase 1 study of the anti-fibroblast growth factor 23 monoclonal antibody, burosumab: Safety and pharmacodynamics in adults with x-linked hypophosphatemia.. J Bone Miner Res..

[19] Insogna KL, Rauch F, Kamenick#x00FD; P (2019). Burosumab improved histomorphometric measures of osteomalacia in adults with x-linked hypophosphatemia: A phase 3, single-arm, international trial.. J Bone Miner Res..

[20] Arango Sancho P (2020). Complications of phosphate and vitamin d treatment in x-linked hypophosphataemia.. Adv Ther..

[21] NICE. (2025). Burosumab for treating X-linked hypophosphataemia in children and young people: Highly specialised technologies guidance HST8.. http://www.nice.org.uk/guidance/hst8.

[22] EMA. Summary of Product Characteristics -CRYSVITA.. http://www.ema.europa.eu/en/documents/product-information/crysvita-epar-product-information_en.pdf.

[23] Minisola S, Peacock M, Fukumoto S (2017). Tumour-induced osteomalacia.. Nat Rev Dis Primers..

[24] Minisola S, Fukumoto S, Xia W (2023). Tumor-induced osteomalacia: A comprehensive review.. Endocr Rev..

[25] Peacock M (2021). Phosphate metabolism in health and disease.. Calcif Tissue Int..

[26] Shimada T, Hasegawa H, Yamazaki Y (2004). FGF-23 is a potent regulator of vitamin D metabolism and phosphate homeostasis.. J Bone Miner Res..

[27] Kolek OI, Hines ER, Jones MD (2005). 1alpha,25-dihydroxyvitamin D3 upregulates FGF23 gene expression in bone: The final link in a renal-gastrointestinal-skeletal axis that controls phosphate transport.. Am J Physiol Gastrointest Liver Physiol..

[28] Saito H, Maeda A, Ohtomo S-I (2005). Circulating FGF-23 is regulated by 1alpha,25-dihydroxyvitamin D3 and phosphorus in vivo.. J Biol Chem..

[29] Folpe AL, Fanburg-Smith JC, Billings SD (2004). Most osteomalacia-associated mesenchymal tumors are a single histopathologic entity: An analysis of 32 cases and a comprehensive review of the literature.. Am J Surg Pathol..

[30] Folpe AL (2019). Phosphaturic mesenchymal tumors: A review and update.. Semin Diagn Pathol..

[31] Jan de Beur SM, Minisola S, Xia W-B (2023). Global guidance for the recognition, diagnosis, and management of tumor-induced osteomalacia.. J Intern Med..

[32] Colangelo L, Pepe J, Nieddu L (2020). Long-term bone mineral density changes after surgical cure of patients with tumor-induced osteomalacia.. Osteoporos Int..

[33] Walton RJ, Bijvoet OL (1975). Nomogram for derivation of renal threshold phosphate concentration.. Lancet..

[34] Feng J, Jiang Y, Wang O (2017). The diagnostic dilemma of tumor induced osteomalacia: A retrospective analysis of 144 cases.. Endocr J..

[35] Rayamajhi SJ, Yeh R, Wong T (2019). Tumor-induced osteomalacia -current imaging modalities and a systematic approach for tumor localization.. Clin Imaging..

[36] Jiang Y, Hou G, Cheng W (2020). Performance of 68Ga-DOTA-SST PET/CT, octreoscan SPECT/CT and 18F-FDG PET/CT in the detection of culprit tumors causing osteomalacia: A meta-analysis.. Nucl Med Commun..

[37] Jing H, Li F, Zhuang H (2013). Effective detection of the tumors causing osteomalacia using [Tc-99m]-HYNIC-octreotide (99mTc-HYNIC-TOC) whole body scan.. Eur J Radiol..

[38] Imanishi Y, Ito N, Rhee Y (2021). Interim analysis of a phase 2 open-label trial assessing burosumab efficacy and safety in patients with tumor-induced osteomalacia.. J Bone Miner Res..

[39] Jan de Beur SM, Miller PD, Weber TJ (2021). Burosumab for the treatment of tumor-induced osteomalacia.. J Bone Miner Res..

